# NeoDoppler: New ultrasound technology for continuous cerebral circulation monitoring in neonates

**DOI:** 10.1038/s41390-019-0535-0

**Published:** 2019-08-12

**Authors:** Sigrid Dannheim Vik, Hans Torp, Turid Follestad, Ragnhild Støen, Siri Ann Nyrnes

**Affiliations:** 1 Department of Circulation and Medical Imaging, Norwegian University of Science and Technology (NTNU), Trondheim, Norway; 2 Children’s Clinic, St. Olavs Hospital, Trondheim University Hospital, Trondheim, Norway; 3 Department of Public Health and Nursing, Norwegian University of Science and Technology (NTNU), Trondheim, Norway; 4 Department of Clinical and Molecular Medicine, Norwegian University of Science and Technology (NTNU), Trondheim, Norway

## Abstract

**Background:**

There is a strong need for continuous cerebral circulation monitoring in neonatal care, since suboptimal cerebral blood flow may lead to brain injuries in preterm infants and other critically ill neonates. NeoDoppler is a novel ultrasound system, which can be gently fixed to the anterior fontanel and measure cerebral blood flow velocity continuously in different depths of the brain simultaneously. We aimed to study the feasibility, accuracy, and potential clinical applications of NeoDoppler in preterm infants and sick neonates.

**Method:**

Twenty-five infants born at different gestational ages with a variety of diagnoses on admission were included. The probe was placed over the anterior fontanel, and blood flow velocity data were continuously recorded. To validate NeoDoppler, we compared the measurements with conventional ultrasound; agreement was assessed using Bland–Altman plots.

**Results:**

NeoDoppler can provide accurate and continuous data on cerebral blood flow velocity in several depths simultaneously. Limits of agreement between the measurements obtained with the two methods were acceptable.

**Conclusion:**

By monitoring the cerebral circulation continuously, increased knowledge of cerebral hemodynamics in preterm infants and sick neonates may be acquired. Improved monitoring of these vulnerable brains during a very sensitive period of brain development may contribute toward preventing brain injuries.

## Introduction

Low cerebral blood flow (CBF) and fluctuations in systemic blood flow in combination with impaired cerebral autoregulation may lead to brain injuries in preterm infants and other critically sick neonates.^1,2^ These brain injuries are associated with high risk for life-long disabilities, such as cerebral palsy, mental retardation, and behavioral problems.^3,4^ Currently, clinicians have to rely on indirect measurements such as assessment of cardiovascular status, including blood pressure, heart rate (HR), capillary refill, lactate, urine production, and echocardiographic measurements, to optimize CBF.^5,6^ However, the definition of systemic hypotension and the criteria for treatment of systemic hypotension to prevent low cerebral perfusion is not clear.^5,7,8^

Although the focus has changed during the past years from assessment of systemic blood pressure as an indicator of end-organ perfusion to directly assessing end-organ perfusion,^9^ the optimal method for direct monitoring of CBF in neonates is lacking. Near infrared spectroscopy (NIRS) enables continuous, non-invasive monitoring of brain oxygenation. However, NIRS only indirectly reflects CBF and is not established in clinical practice.

There is a strong need for a method that can monitor CBF continuously and directly. By identifying low CBF and high-risk alterations, medical interventions can be optimized to protect these vulnerable brains. NeoDoppler is a new ultrasound system with a small, lightweight ultrasound probe that can be gently fixed to the patient’s fontanel and continuously measure CBF velocities at different depths of the brain simultaneously. We aimed to study the feasibility, accuracy, and potential clinical applications of NeoDoppler in premature babies and sick neonates.

## Material and methods

### Participants and design

In this feasibility study, a purposive sample of 25 patients admitted to the Neonatal Intensive Care Unit (NICU) at St. Olavs Hospital, Trondheim University Hospital, Trondheim, Norway, between October 13, 2017 and October 14, 2018 were enrolled to ensure a variety of gestational ages (GAs), birth weights (BWs), and clinical conditions. Infants in critical condition or undergoing invasive procedures were not included. The NeoDoppler probe was placed over the anterior fontanel for a duration of 3–4 h.

### Clinical data

Information regarding GA, BW, postmenstrual age, postnatal age in hours at inclusion, clinical diagnosis, medical treatment, and breathing support at inclusion was collected from the medical records.

### The NeoDoppler system

**Fig. 1:**
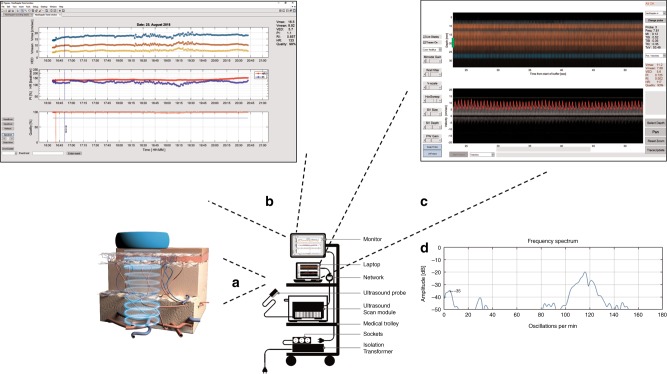
The NeoDoppler research set-up. **a** illustrates the NeoDoppler probe. The NeoDoppler probe transmits plane waves and simultaneously receives echoes from blood vessels at different depths covered by the ultrasound beam. **b** demonstrates trend curves obtained during a period of 4 h in a preterm infant. The upper panel shows the trend curves for maximum (Vmax, blue), mean (Vmean, red), and end-diastolic velocity (VED, yellow); panel in the middle shows pulsatility index (PI, blue) and heart rate (HR, red); the lower panel shows the quality of the measurements (red). **c** shows depth-vs-time color M-mode (upper panel), which represents blood flow in several vessels at different depths for a duration of 30 s. The lower panel shows pulsed-wave Doppler velocity waveforms for a specific depth. The green marker in the upper panel, representing the sample volume (SV), indicates the depth where the curve in the lower panel is obtained. One can change the size and position of the sample volume with controls labeled SV depth and SV size. The SV width can be set to cover the artery of interest in each patient. **d** shows the frequency spectrum where the oscillations in the pulsed-wave Doppler spectrum are represented. The frequency scale, *x* axis, is presented as oscillations/min and the corresponding amplitude in decibels, *y* axis, is relative to the mean value of the velocity trace. In this case, the HR of the infant contributes to the major peak at 115/min. The respiratory frequency contributes to the peak at 30/min. The peak at the low frequencies, about 5–7/min, represents the slow observed oscillations in the velocity spectrum, in this case with an amplitude of −35 dB

NeoDoppler is a non-invasive ultrasound Doppler system, recently developed by the ultrasound group at the Norwegian University of Science and Technology (NTNU) in Trondheim, Norway. The research set-up consists of the NeoDoppler probe (Fig. 1a), a scanner (Manus EIM-A, Aurotech Ultrasound AS, Tydal, Norway), and a user interface with display (Fig. 1b, c).

The NeoDoppler probe operates at the frequency of 7.8 MHz and the transmitted beam covers a wide area with cylindrical shape of diameter 1 cm, in a depth range from 3 to 35 mm. CBF velocities are measured in 41 parallel slices with spacing 0.78 mm at increasing distances from the probe. The depth interval intersecting with each artery depends on the position and orientation of the artery. The prototype probe used in this study was equipped with an 8 MHz transducer, manufactured by Imasonic SAS, France. The scanner component includes electronics for an ultrasound transmitter, receiver, real-time processing, and transmission of received echo data via a network cable to the PC.

### The research user interface

The pulse echo signals from the scanner are transferred to the display unit and processed in real time to produce a color M-mode display and a Doppler spectrogram. The user interface includes two screens, one provides the real-time Doppler signals in multiple depths simultaneously (Fig. 1c). These signals include depth vs time color M-mode, representing blood flow in several vessels at different depths, and the corresponding pulsed-wave Doppler curves from one specific depth. The M-mode display shows the direction of flow (red or blue) and the intensity of the signal (color brightness) of each blood vessel intersecting with the ultrasound beam. The depth interval for each vessel is not directly related to the vessel diameter but depends on the position and orientation of the vessel. An example: A straight artery passing through the center of the ultrasound beam, with 45-degree angle, will give a depth interval of 1 cm (equal to the beam diameter).

If the wide sound beam at a specific depth picks up several arteries, the maximum velocity trace will represent the vessel with the highest velocity. Signals from veins and arteries can be distinguished from each other by the Doppler spectrum. No angle correction is performed. The sample volume indicates the depth where the pulsed-wave Doppler curves is obtained. Based on the velocity measurements and the quality of the measurements, trend curves are visualized on a separate screen (Fig. 1b). The quality of the measurements is calculated for each heartbeat based on the correlation coefficient between the current and the previous heartbeat, graded from 0 to 100%.

Simple commands such as start/stop, selection of the preset time intervals, and registration of events can be done during recording. The preset time intervals can be adjusted depending on the clinical situation of the infant and to keep the duration of the exposure of ultrasound waves to a minimum. Adjustments such as selection of depth, sample volume width, gain, velocity scale, and wall filter can be performed during the recordings or by post-processing. Tracing of the Doppler velocity spectrum is performed by the software after each interval of recordings and the corresponding velocity measurements, including pulsatility index (PI); resistance index (RI); HR; maximum (Vmax), mean, and end-diastolic velocities, are continuously displayed. In addition, the quality of the velocity measurements is displayed after each time interval of recording. The tracing can optionally be turned off during the recordings or during post-processing.

Observed oscillations in the pulsed-wave Doppler velocity curves are demonstrated in a corresponding frequency spectrum (Fig. 1d). The frequency is given as oscillations/min. The more oscillations at the same frequency, the higher and narrower the amplitude at that specific frequency. An in-house software developed in MATLAB (MathWorks ® R2017a) performs the data processing.

### Fixation and positioning of the NeoDoppler probe

To enable continuous recording over time, a specially designed housing integrated in a soft hat fixated the probe (Supplemental Fig. S1). The probe housing was developed in cooperation with a product design company (Inventas, Trondheim, Norway). The ultrasound signal was achieved by placing the probe over the anterior fontanel. The soft hat can be combined with other medical equipment such as fixation of invasive or non-invasive breathing support and electrodes for amplitude integrated electroencephalography.

### In vitro and in vivo validation

A flow phantom (Doppler Ultrasound Flow Simulator, Model 069; Computerized Imaging Reference Systems, Inc (CIRC)) was used for the in vitro validation comparing NeoDoppler with a conventional ultrasound system with a 9L linear probe (Vivid E9, GE Ultrasound, Horten, Norway), with frequencies set to 6.6 and 6.7 MHz, respectively. To validate the NeoDoppler output in vivo, velocity measurements were obtained with the conventional ultrasound system with a 12S phased array probe (GE Healthcare, Milwaukee, WI) with frequencies set to 7.8 and 7.7 MHz (NeoDoppler and conventional ultrasound probe, respectively). Both probes were held manually over the anterior fontanel. The vessels compared were at the same depth and had similar Doppler velocity waveforms. We compared the average of ten pulsed-wave Doppler curves obtained with standard ultrasound with the average of Doppler curves obtained during 7 s of recording with NeoDoppler. The measurements for both the in vivo and in vitro validation included comparison of Vmax, with additional comparison of PI in vivo.

### Safety and user friendliness

The acoustic output of the NeoDoppler was measured using a water-tank and a computer-controlled hydrophone in a laboratory at the Department of Circulation and Medical Imaging of NTNU before it was applied on the infants. The absolute upper limit for thermal index (TI) set for the NeoDoppler system was 0.7 degrees, in accordance with the upper limit in neonatal transcranial ultrasound in cases where there is no restriction in exposure time.^10^ In order to minimize potential temperature increase, each interval of recording lasted 7 s followed by a 10 s pause (the first six patients) or 30 s recordings with pauses of 30 s (in the last 19 patients). The time interval was changed during the study to be able to better capture the observed slow periodic flow oscillations. The skin was examined after removal of the probe to assess for any local adverse effects from the probe. The parents and nurses were given a questionnaire for feedback on the probe and the fixation system.

### Data and statistical analyses

From each patient, periods with velocity measurements of good quality over time (defined as >80%) in the same vessel were used for analyses. Analysis of the velocity measurements obtained with conventional ultrasound was performed with Echopac^TM^. The statistical analyses were performed using IBM SPSS Statistics version 21.0 (IBM, New York, USA). Agreement between NeoDoppler and conventional ultrasound was assessed using Bland–Altman plots. The limits of agreement were set to mean difference ±1.96 × SD. Paired sample *t* test was used to compare mean difference between NeoDoppler and conventional ultrasound. The standard deviation (SD) of the residuals from a linear regression model with a linear trend over time was used to estimate the variability in PI over time.

The Regional Committee for Medical and Health Research Ethics, REC Central (Reference 2017/314) and The Norwegian Directorate of Health (Reference: 17/15181-11) approved the study. Written informed consent was obtained from the parents.

## Results

### Patient data

Twenty-five infants, 11 girls and 14 boys, were included during a period of 12 months. One extremely preterm infant, born at GA 24.9 weeks was monitored twice (at 18 h of age and at day 36 of life) and is presented as two inclusions. Fourteen infants were preterm with median GA 30.9 weeks (range 23.1–36.0) and median BW 1525 g (range 500–3650 g). Three of the preterm infants were on mechanical ventilation, and six were on non-invasive ventilatory support at the time of assessment. The remaining 11 infants were born at term with median BW 4040 g (range 3300–4415 g). Of these, three were treated with therapeutic hypothermia for neonatal encephalopathy and received mechanical ventilation, one had undergone surgery and was on mechanical ventilation, and six had been admitted owing to suspected infection or wet-lung, one of whom was on non-invasive ventilatory support. One infant was a healthy baby assessed in the maternity unit. Median postnatal age of NeoDoppler assessment was 1.8 days (range 0.6–36 days).

### In vitro and in vivo validation

**Fig. 2:**
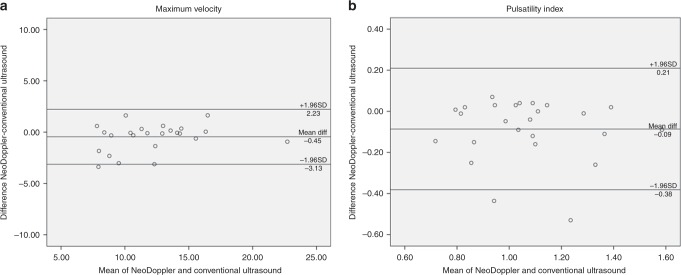
Agreement between NeoDoppler and conventional ultrasound for maximum velocity (**a**) and pulsatility index (**b**) in all study subjects

The flow measurements in the flow phantom with NeoDoppler and conventional ultrasound showed good agreement for the maximum velocity (Vmax 22.8 and 22.96 cm/s, respectively). Bland–Altman plots (Fig. 2) illustrate the agreement between measurements of Vmax and PI obtained with NeoDoppler and conventional ultrasound in vivo. The mean Vmax measured with NeoDoppler was 12.42 cm/s (SD: 4.53), and the mean Vmax measured with conventional ultrasound was 12.87 cm/s (SD: 4.06), yielding a mean difference between the measurements of −0.45 cm/s (SD 1.37). The 95% limits of agreement were −3.13 and 2.23 cm/s (Fig. 2a). The mean difference between Vmax obtained with the two methods did not differ significantly from zero (*p* = 0.113).

The mean PI measured with NeoDoppler was 1.02 (SD: 0.22), and the mean PI measured with conventional ultrasound was 1.11 (SD: 0.23), yielding a mean difference of −0.09 (SD: 0.15) between the measurements. No obvious trend in the data was observed and the 95% limits of agreement were −0.38 and 0.21 (Fig. 2b). The mean difference between PI obtained with the two methods differed significantly from zero (*p* = 0.008), indicating slightly lower values with NeoDoppler compared to conventional ultrasound.

### Feasibility

The NeoDoppler was attached to the anterior fontanel for a median duration of 3:00 h (range 0:31–6:59 h) in 24 patients. One infant was excluded from the continuous flow monitoring due to an intravenous line placed close to the anterior fontanel. In two infants, only sporadic good quality measurements from the same vessel were achieved. From 22 infants, periods with good quality velocity measurements from one vessel over time were selected, the median duration of the selected periods was 2:06 h (range 0:10–6:04 h). Vessels at two different depths could be measured simultaneously for a median duration of 1:02 h (range 00:16–3:20 h) in 6 infants, whereas vessels from three different depths could be measured simultaneously for a median duration of 2:11 h (range 1:38–6:04 h) in 3 infants.

### Selected patient examples

**Fig. 3:**
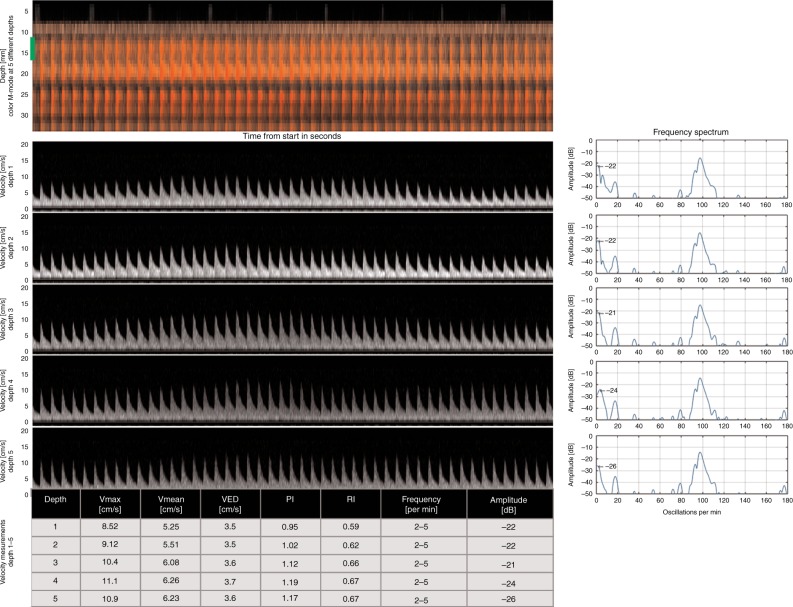
Velocity measurements at different depths simultaneously in a term infant with corresponding frequency spectrums. The figure shows 30 s selected from 6 h of recording with NeoDoppler showing Doppler curves in five different vessels at different depths in a term infant treated with therapeutic hypothermia for hypoxic–ischemic encephalopathy and on mechanical ventilation. The corresponding frequency spectrums at each depth are demonstrated to the right and each of them shows one major peak corresponding to the heart rate of the infant (100/min) and one peak at low frequency (2–5 oscillations/min), which represent the observed slow periodic flow oscillations seen in the Doppler velocity curves. Depths in cm, 1: 1.2–1.6, 2: 1.7–2.2, 3: 2.4–2.7, 4: 2.7–3.1, 5: 3.1–3.5

**Fig. 4:**
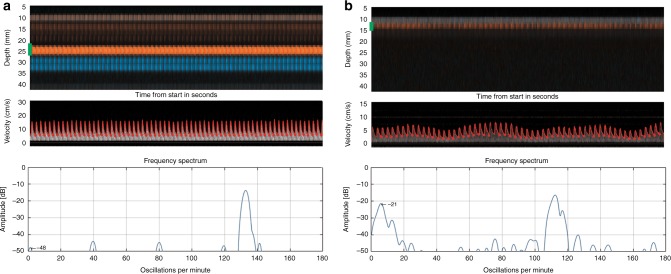
Observed oscillations in the Doppler velocity curves during 30 s of recording with corresponding frequency spectrums. The green marker in the color M-mode represents the sample volume at the depth where the Doppler velocity spectrum is obtained. **a** Cerebral blood flow without oscillations in a vessel at depth 2.2–2.6 cm in one late preterm infant with neonatal sepsis and intestinal perforation, recorded with NeoDoppler postoperatively at the time when the infant was considered hemodynamically unstable. The corresponding frequency spectrum has one major peak at about 135/min corresponding to the heart rate of the infant. There are no other major peaks in the frequency spectrum. **b** Cerebral blood flow with oscillations in a vessel at depth 1.2–1.5 cm in a term infant with infection. The corresponding frequency spectrum has one peak corresponding to the heart rate of the infant (110/min) and one peak at low frequency (5–7/min), which represents the observed slow periodic flow oscillations seen in the Doppler velocity curves

**Fig. 5:**
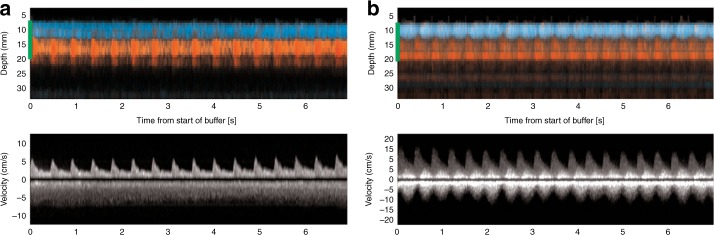
Simultaneous velocity measurements of arterial and venous signals. The green marker in the color M-mode, representing the sample volume, indicates the depth where the Doppler velocity curves are obtained and covers both the arterial and the venous signals so that both signals are represented in the Doppler velocity curves. **a** Measurements of 7 s where the arterial (red) and venous (blue) signals can be measured simultaneously in a term infant with transient tachypnea of the newborn. The pulsed-wave Doppler signal demonstrates no pulsation in the venous signal. **b** Measurements of 30 s where arterial (red) and venous (blue) signals are measured simultaneously in a late preterm infant with gastroschisis, recorded with NeoDoppler postoperatively. The pulsed-wave Doppler signal demonstrates venous pulsation

**Fig. 6:**
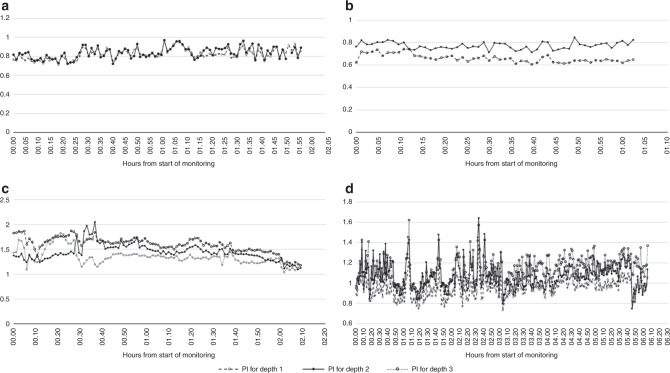
Continuous recording of the pulsatility index (PI) at different depths simultaneously. **a** Two hour recording in one late preterm infant. Depth 1 (1.5–2 cm): Mean PI 0.82 (SD 0.06). Depth 2 (2.5–3.1 cm): Mean PI 0.84 (SD 0.06). **b** One hour recording in one very preterm infant. Depth 1 (1–1.5 cm): Mean PI 0.66 (SD 0.03). Depth 2 (1.5–2 cm): Mean PI 0.77 (SD 0.03). **c** Two and a half hour recording in a term infant treated with therapeutic hypothermia for neonatal encephalopathy and on mechanical ventilation. Depth 1 (1.7–2.1 cm): Mean PI 1.35 (SD 0.13). Depth 2 (2.7–3.5 cm): Mean PI 1.44 (SD 0.16). Depth 3 (3.9–4.2 cm): Mean PI 1.6 (SD 0.10). **d** Six hour recording in a term infant treated with therapeutic hypothermia for neonatal encephalopathy and on mechanical ventilation. Depth 1 (1.3–1.9 cm): Mean PI 0.96 (SD 0.09). Depth 2 (1.9–2.2 cm): Mean PI 1.07 (SD 0.13). Depth 3 (2.3–3 cm): Mean PI 1.14 (SD 0.12)

We selected relevant case examples that demonstrate new features obtained with NeoDoppler. First, we demonstrate Doppler curves representing velocity measurements in five different depths simultaneously during 30 s of recording and the corresponding color M-mode at depths ranging from 1.2 to 3.5 cm (Fig. 3). The oscillations at different depths appear to have similar patterns and similar Doppler velocity curves and frequency spectrums. Figure 4 shows Doppler velocity curves during 30 s of recording and frequency spectrums from one hemodynamically unstable and one hemodynamically stable infant (Fig. 4a, b, respectively), illustrating differences in oscillation patterns. In both cases, the frequency spectrums have one major peak at the frequency corresponding to the HR of the infant (135 and 110/min, respectively). In the hemodynamically unstable infant, there are no other major peaks in the frequency spectrum, indicating no major oscillations during these 30 s of recording. This was also seen in the Doppler velocity curves. The hemodynamically stable infant has one peak at low frequency (5–7 oscillations/min, corresponding to around 0.1 Hz), which represents the observed slow periodic flow oscillations seen in the Doppler velocity curves. Further, the arterial and venous signals could be measured simultaneously, exemplified in Fig. 5, showing one example of no venous pulsation (Fig. 5a) and one example of venous pulsation (Fig. 5b). Finally, we present continuous recordings of the PI at different depths simultaneously for a duration of 1–6 h in four patients (Fig. 6a–d), where the PI seems to be parallel at different depths in the same patient. The mean PI over time in all patients ranged from 0.66 to 1.69 while the SD, representing the variability in PI over time, ranged from 0.03 to 0.16.

### Safety and user friendliness

Values of thermal and mechanical indices were continuously displayed during recordings and were within the recommended limits.^10^ Mechanical index (MI) ranged from 0.1 to 0.13 at the skin surface, the highest value dropping to 0.076 at 2 cm depth. The TI for bone and cranial bone ranged from 0.17 to 0.42, whereas the TI for soft tissue ranged from 0.25 to 0.61. The temperature increase was up to 0.61 degrees at the skin surface, dropping to 0.2 degrees at 2 cm depth with continuous ultrasound exposure. With the intermittent recordings, 30 s on and 30 s off, the maximum temperature increase was reduced to 0.3/0.1 degrees, respectively.

Twenty-one nurses and 20 parents returned the questionnaire on patient comfort and feasibility of the fixation system in combination with nursing procedures and skin-to-skin care with the parents. Five nurses reported skin problems such as redness or skin impression related to the NeoDoppler fixation, but none were serious. None of the nurses reported that NeoDoppler affected medical treatment or nursing and none of the parents or nurses reported that NeoDoppler interrupted skin-to-skin care. Neither the nurses nor the parents found any sign of pain displayed by the infants during recording with NeoDoppler. Except for one of the nurses, none of the respondents perceived that the infants felt discomfort. All of the nurses and parents felt well informed about NeoDoppler and understood the potential future benefits of this new technology. Both nurses and parents provided suggestions on the properties and the fixation of the probe. These suggestions have been incorporated in the future development of the system.

## Discussion

In this feasibility study, we have shown that a new ultrasound system for monitoring cerebral circulation provides accurate and continuous data on CBF velocity in several depths simultaneously in neonates at different GAs and with different clinical conditions. Measurements obtained with NeoDoppler showed good agreement with measurements obtained with conventional ultrasound.

### Methodological strengths and limitations of NeoDoppler

The main strength of this new technology is that it enables continuous monitoring of cerebral circulation. In contrast to conventional technology, NeoDoppler requires minimal training of the examiner. Doppler signals from several vessels can easily be obtained by placing the probe over an open fontanel. A potential limitation is that we do not know exactly which vessel the measurements are obtained from. However, NeoDoppler enables assessment of blood flow velocity in several vessels simultaneously, both in the small and large network of blood vessels within the area of the ultrasound beam. This may be considered a strength compared with conventional ultrasound where assessment of cerebral circulation is mainly based on measurements in large vessels.^11,12^ Our preliminary experience indicates that following a trend over time may be more clinically relevant than conventional snapshot velocity measurements in one specified artery to assess cerebral circulation. Finally, NeoDoppler appeared to be clinically feasible and well tolerated as a non-invasive, bedside tool for continuous monitoring.

The main limitation of the present system is the potential for dislocation of the probe, which may reduce the signal quality. To account for this, a quality indicator is displayed, and the system removes signals with suboptimal quality. Further development of the probe fixation may address this challenge, and input from nurses and parents ensure continuous upgrading of the probe housing. The goal is a firmly fastened probe that does not disturb the infant, the contact between the infant and the parents, medical procedures, or nursing.

NeoDoppler measured a slightly lower PI than conventional pulsed-wave Doppler in vivo. This can be explained by different beam-flow angles with the two methods. In standard ultrasound, optimization of the beam-flow angle can be navigated by two-dimensional image, while NeoDoppler are only guided by M-mode display. For the PI, we believe that the trend over time is the most important, and small differences in absolute measurements is probably not of clinical significance.^13^

### Safety

Concerns on safety of long-term ultrasound and Doppler on the brain of the fetus and newborn have been raised.^14^ However, diagnostic ultrasound has an excellent safety record when the exposure is within the recommended limits.^10^ For the NeoDoppler probe, the TI decreased from 0.61 at the skin surface to 0.07 at 4 cm depth. In contrast to conventional UL, the NeoDoppler plane wave probe creates the highest TI at the surface and not in the brain tissue. However, it is good clinical practice to limit exposure times whenever possible, and intermittent recordings were therefore chosen. MI at the skin surface in our studies ranged from 0.1 to 0.13, which is also in the lower range of the recommended limits.^10^ In future use of NeoDoppler, the scanning protocols may be adapted to the clinical situations where intermittent recordings over time as described often will be sufficient. During procedures, a continuous mode can be selected and then adjusted again to conventional intermittent modus when procedures are completed.

### New features obtained with NeoDoppler

NeoDoppler can provide information on velocity measurements over time. Three previous studies have reported on continuous monitoring with pulsed-wave Doppler measurements in neonates using conventional ultrasound.^15–17^ It was demonstrated already in 1990 that one specific artery could be followed for several hours with conventional pulsed-wave Doppler, enabling assessment of normal and pathological variations in velocities over time and identification of acute changes in blood flow velocities caused by medication, ventilation, or clinical derangement.^15^ However, such continuous conventional Doppler measurements have been unsuitable for use in clinical practice. In contrast, NeoDoppler provides a feasible method for bedside monitoring for several hours or even days. Currently, NIRS can monitor cerebral oxygenation continuously, and the variations in cerebral oxygenation is used as an indicator of cerebral circulation. Since the first NIRS publication in 1985,^18^ NIRS has been considered promising^9,19^ and has contributed to increased knowledge about cerebral hemodynamics.^19,20^ However, NIRS is still not established as standard monitoring equipment to assess end-organ perfusion, and evidence of improved short- and long-term outcomes with the use of NIRS is still lacking.^20–22^ Monitoring of cerebral activity with electroencephalography is frequently used in NICUs, and a recent study showed association between seizure activity and an increase in CBF, measured by ultrafast Doppler.^23^ Future research will demonstrate the role for NeoDopppler in assessment of cerebral circulation during seizure activity. Finally, transcranial Doppler ultrasound has been utilized for continuous cerebral monitoring in adult and pediatric patients.^24,25^ However, this technique is hampered by several limitations and is not suitable in a bedside neonatal clinical setting.

A cerebral protective effect of short timescale hemodynamic variability has been suggested^26^ and is in accordance with our findings of lack of oscillations in very sick and unstable infants. Low frequency oscillations of several physiological variables of the cardio-vascular system is a well-known phenomenon and it is thought to be driven by mechanisms related to cerebral autoregulation.^27^ However, low frequency data collection of physiologic monitoring signals may not accurately reflect the variability and complexity of these signals or the patient’s clinical state.^28^ The NeoDoppler system has a high temporal resolution (frame rate 300/s), and data sampling can be synchronized with, for example, cardiac measurements and blood pressure. This can increase the pathophysiologic understanding of cerebral autoregulation and disease processes.

In preterm infants, fluctuations in CBF is a major factor contributing to the risk of intraventricular hemorrhage (IVH).^29^ However, there is increasing evidence that arterial blood pressure is an inadequate measure of cerebral perfusion pressure and CBF in neonates.^30^ Furthermore, a recent study using continuous NIRS monitoring^31^ found that cerebral oxygenation was lower in infants who developed IVH, and loss of cerebrovascular reactivity was present just before or during the hemorrhage. High-grade fluctuations in the perfusion waveform of the internal cerebral vein has also been shown to be associated with a high incidence of IVH.^32^ With the ability to measure arterial and venous signals simultaneously, NeoDoppler provides a novel tool for bedside monitoring of cerebral hemodynamics, so that early signs of instability can be addressed and, potentially, contribute to reduced incidence of IVH.

We found that oscillations and the Doppler velocity waveforms from different blood vessels at different depths had similar patterns. This is in line with previous studies demonstrating a good correlation between flow indices in the anterior and in the middle cerebral artery,^15,33^ as well as between the right and left great vessels.^34^ These findings support that identifying exactly the same vessel is not essential to assess the cerebral circulation. NeoDoppler can therefore be feasible for non-expert users, who can focus only on capturing a high-quality flow signal within the broad ultrasound beam.

NeoDoppler can continuously monitor flow indices such as PI and RI, which reflect vascular resistance. Whether or not flow indices are good indicators to assess cerebral hemodynamics remains controversial.^11,33,35,36^ In neonatology, RI has been used to predict outcome in infants with neonatal encephalopathy. A low RI (<0.55) has been associated with poor neurological outcome,^37^ but this association appears weaker during therapeutic hypothermia.^38^ With NeoDoppler, continuous monitoring of changes in vascular resistance will be possible and may reveal new information about CBF before, during, and after therapeutic hypothermia in infants with neonatal encephalopathy. Doppler measurements can also be used to detect abnormal Doppler velocity waveforms, such as decreased or reversed diastolic flow in cerebral arteries caused by hemodynamically significant patent ductus arteriosus.^36,39^ In the future, continuous monitoring with NeoDoppler may contribute to optimize medical treatment in infants with this condition.

To what extent velocity measurements obtained with Doppler represents CBF is controversial. The diameter of the arteries constantly varies as a response to physiological changes, thus affecting the velocity measurements. Some studies have reported good correlation between velocity measurements and CBF,^40–42^ whereas other studies indicate that velocity measurements only reflect CBF if the diameter of the artery is kept constant.^43–46^ Noori et al. used velocity as a surrogate for CBF but could not exclude that changes in the diameter may affect only the velocity without affecting the CBF. However, they found correlation between velocity measurements and NIRS, which was further correlated with changes in partial pressure of carbon dioxide and oxygen in blood.^41,42^ This may support the idea that velocity measurements obtained with Doppler can be good indicators of CBF.

## Conclusion

A new ultrasound system, NeoDoppler, provides accurate and continuous recording of Doppler signals in several depths of the brain simultaneously through an open fontanel and is feasible in preterm and sick infants. NeoDoppler has the potential to become a standard bedside monitoring system to guide hemodynamic management of these patients. Further studies are required to increase our knowledge about how the different NeoDoppler outputs can be used to identify and correct clinically relevant cerebral hemodynamic instability during a very sensitive period of brain development.

## Supplementary information

Supplementary Figure
