# Supplementary figures and images for: NeoDoppler: New ultrasound technology for continuous cerebral circulation monitoring in neonates
Source: Pediatr Res. 2019 Aug 12;87(1):95–103. doi: 10.1038/s41390-019-0535-0 (PMC6960092; doi:10.1038/s41390-019-0535-0)

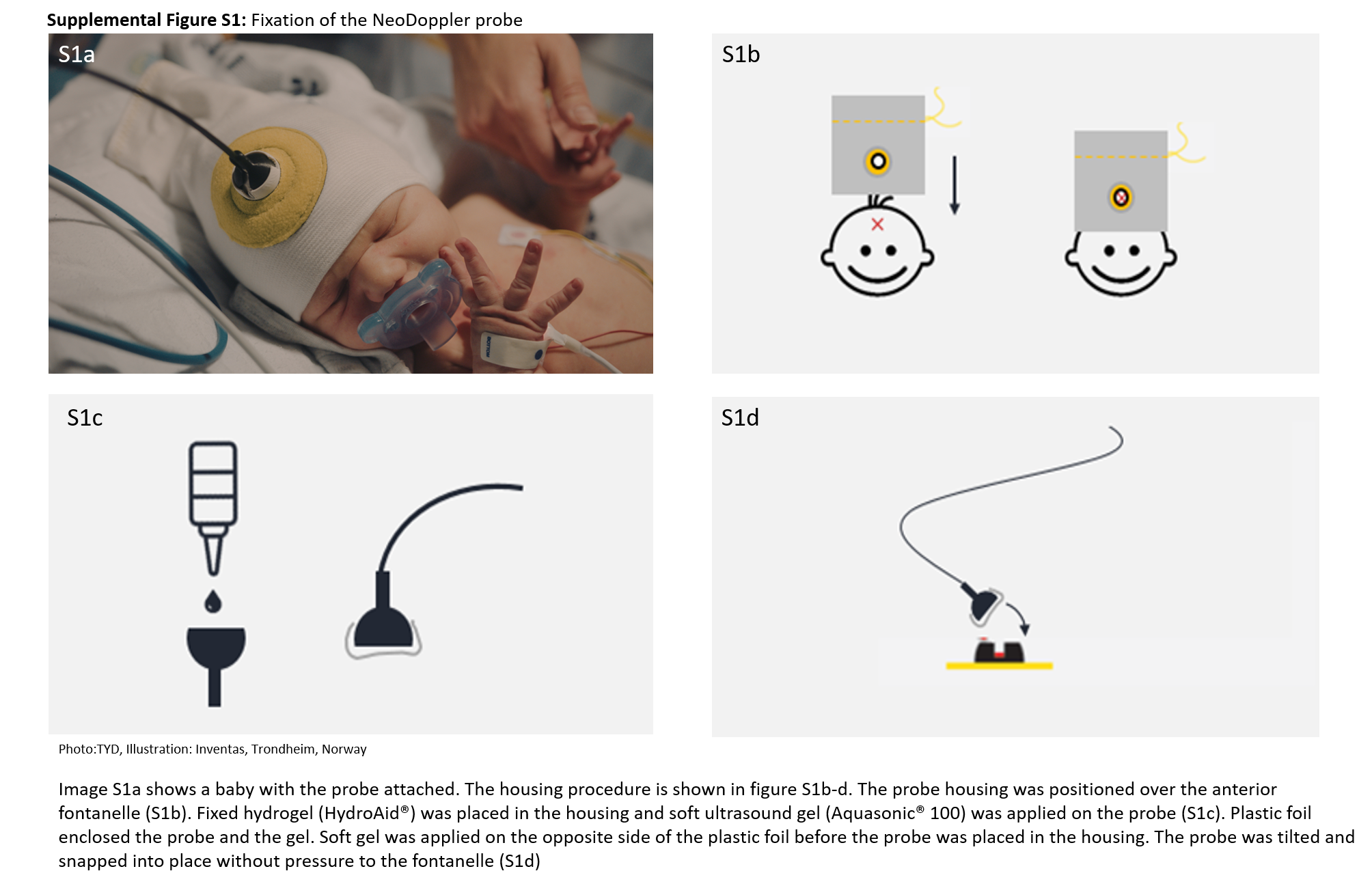

Supplement: Supplementary file 1 — Supplementary Figure [file 41390_2019_535_MOESM1_ESM.tif]
